# Reviewer acknowledgement 2015

**DOI:** 10.1186/s13030-016-0053-9

**Published:** 2016-02-18

**Authors:** Gen Komaki, Mutsuhiro Nakao, Shin Fukudo

**Affiliations:** International University of Health and Welfare, School of Health Sciences, Fukuoka, 831-8501 Japan; Teikyo University Graduate School of Public Heath, Tokyo, 173-8605 Japan; Tohoku University Graduate School of Medicine, Sendai, 980-8575 Japan

## Abstract

The *BioPsychoSocial Medicine* editorial team would like to thank all reviewers who have contributed to the journal in Volume 9 (2015).

**Tetsuya Ando**

Japan

**Shuji Aou**

Japan

**Kashif Aziz**

Pakistan

**Hans-Christian Deter**

Germany

**Shin Fukudo**

Japan

**Mikihiko Fukunaga**

Japan

**Hirokazu Furukawa**

Japan

**Yusuke Hada**

Japan

**Masahiro Hashizume**

Japan

**Hirono Ishikawa**

Japan

**Yuko Ishizaki**

Japan

**Motoyori Kanazawa**

Japan

**Kenji Kanbara**

Japan

**Michiko Kano**

Japan

**Hiroe Kikuchi**

Japan

**Masayo Kojima**

Japan

**Hiroaki Kumano**

Japan

**María Torres Lacomba**

Spain

**Mariko Makino**

Japan

**Astrid Müller**

Germany

**Masanori Munakata**

Japan

**Shinichiro Nagamitsu**

Japan

**Jun Nagano**

Japan

**Toshihiko Nagata**

Japan

**Mutsuhiro Nakao**

Japan

**Shinobu Nomura**

Japan

**Yuko Odagiri**

Japan

**Mariko Ogawa**

Japan

**Kyoko Ohashi**

United States

**Takakazu Oka**

Japan

**Hirokuni Okumi**

Japan

**Yoshifumi Okura**

Japan

**Annelieke Roest**

Netherlands

**Amy Ronaldson**

United Kingdom

**Ken Shimizu**

Japan

**Hideaki Soya**

Japan

**Nobuyuki Sudo**

Japan

**Nagisa Sugaya**

Japan

**Shu Takakura**

Japan

**Takeaki Takeuchi**

Japan

**Masato Takii**

Japan

**Tetsushi Tamekazu**

Japan

**Yoshihisa Wada**

Japan

**Jong Min Woo**

South Korea

**Kazufumi Yoshihara**

Japan

**Kazuhiro Yoshiuchi**

Japan

**Stephan Zipfel**

Germany

